# The effect of Kuntai capsule on ovarian function in cisplatin-induced premature ovarian insufficiency rats

**DOI:** 10.3389/fendo.2022.1097165

**Published:** 2023-01-19

**Authors:** Suiyu Luo, Xiangyan Ruan, Alfred O. Mueck

**Affiliations:** ^1^ Department of Gynecological Endocrinology, Beijing Obstetrics and Gynecology Hospital, Capital Medical University, Beijing Maternal and Child Health Care Hospital, Beijing, China; ^2^ Department for Women’s Health, University Women’s Hospital and Research Center for Women’s Health, University of Tuebingen, Tuebingen, Germany

**Keywords:** Kuntai capsule, cisplatin, premature ovarian insufficiency, rats, protein expression, ovarian function

## Abstract

**Objective:**

This study aims to evaluate the effect of Kuntai capsule on ovarian function in cisplatin-induced premature ovarian insufficiency rats and to explore the mechanism of Kuntai capsule on the ovarian function of rats.

**Methods:**

Seventy-four female Sprague-Dawley rats were used for this study. Eight of the rats were randomly assigned to the Control group. The remaining sixty-six rats were utilized to establish the POI model *via* Cisplatin and then randomly divided into four groups: the model Control group, the Estradiol group, and groups treated with low and high doses of Kuntai capsule. For the 28-day administration, the Control and model Control groups were intragastrically administered with 2.0 mL of 0.9% sodium chloride daily, the Estradiol group with 2.0 mL of Estradiol suspension (0.2mg/kg/d), and the low dose Kuntai capsule group and the high dose Kuntai capsule group with 2.0 mL of Kuntai capsule suspension (0.6g/kg/d, 1.8g/kg/d, respectively). Sex hormone levels, estrous cycle, and ovarian coefficient of the five groups were compared, histological sections analyzed follicle counts, and the protein expressions of growth differentiation factor 9, light chain 3 A-II, and Beclin 1 in the ovarian tissue were detected by Western blotting.

**Results:**

After the 28-day administration, the serum Estradiol and Follicle-Stimulating Hormone levels of the group treated with low dose of Kuntai capsule were not significantly different from the Control group, the serum anti-Müllerian Hormone level of the group treated with high dose of Kuntai capsule was significantly higher than the Estradiol group. The estrous cycle of the group treated with low dose of Kuntai capsule was significantly lower than the model Control group. Regarding ovarian coefficient, resting and growing follicles, growth differentiation factor 9, light chain 3 A-II, and Beclin 1 expression, both Kuntai capsule groups outperformed the model Control group with the statistical difference (*P*<0.05).

**Conclusion:**

Kuntai capsule can improve the estrous cycle and ovarian coefficient of rats with premature ovarian insufficiency, maintain the number of resting and growing follicles, and up-regulate the protein expression of growth differentiation factor 9, light chain 3 A-II, and Beclin 1 of rats’ ovaries.

## 1 Introduction

Premature ovarian insufficiency (POI) is defined as a cessation of ovarian function before the age of 40 years. European Society of Human Reproduction and Embryology (ESHRE) recommends the following diagnostic criteria: amenorrhea or oligomenorrhoea for at least four months and elevated follicle-stimulating hormone (FSH) levels (> 25 IU/L) on two occasions (> 4 weeks apart) (1).

Over the past 20 years, the development of spontaneous POI has been relatively stable, but induced POI is on the rise. Worldwide, over 6.6 million women are diagnosed with cancer each year, and about 10% of them are diagnosed during reproductive age (age < 40); they usually receive aggressive chemotherapy and radiotherapy, which may cause POI and subsequent fertility loss in more than 80% of cases (2–6).

According to the National Cancer Institute, up to 10% to 20% of all cancer patients have been prescribed with Cisplatin and other platinum-based drugs. Cisplatin is a cell cycle non-specific drug that destroys the primordial follicle, induces follicle apoptosis, and reduces the follicle’s pool. Treatment with Cisplatin exceeding 400mg could lead to irreversible ovarian damage (7, 8).

Can Kuntai capsule (a traditional Chinese medicine) preserve or improve patients’ ovarian function after they are treated with Cisplatin? Kuntai capsule is derived from Huanglian Ejiao Decoction, a classic prescription from *Treatise* on *Febrile*, written by Chinese sage Zhang Zhongjing in the early 3rd century. It consists of six herbs for treating ovarian insufficiency: Rehmannia glutinosa, Coptis, Paeonia, Scutellaria, Equus asinus, and Poria cocos.

In this study, Kuntai capsule was used to treat Cisplatin-induced POI rats, and its effect and possible mechanisms on these rats’ ovarian function were preliminarily explored.

## 2 Materials and methods

### 2.1 Experimental design

After ten days of adaptive feeding, seventy-four specific-pathogen-free grade healthy female Sprague-Dawley rats, aged 8-week-old and weighing 180-200g (Animal License: SCXK (Beijing) 2011-0012), were involved in this study. These rats were housed in groups of 3 per cage and maintained under standard laboratory conditions (20-24°C; relative humidity: 50%-70%, light cycles: 10h light, 14h dark; bedding material kept dry).

These rats were then randomly divided into the Control group (n=8) and the POI model group (n=66), the latter intraperitoneally administered with 2ml of Cisplatin Injection (Jiangsu Hansoh Pharmaceutical Co. Ltd., lot number: H20040812, strength: 6ml:30mg) at a dose of 6mg/kg for once a week for two weeks to establish the model (9–13). The POI model group was then randomly divided into four groups: the model Control group (mo Control), the group treated with E2 (E_2_), and the groups treated with low and high doses of Kuntai capsule (KTC-L, KTC-H).

**Figure 1:**
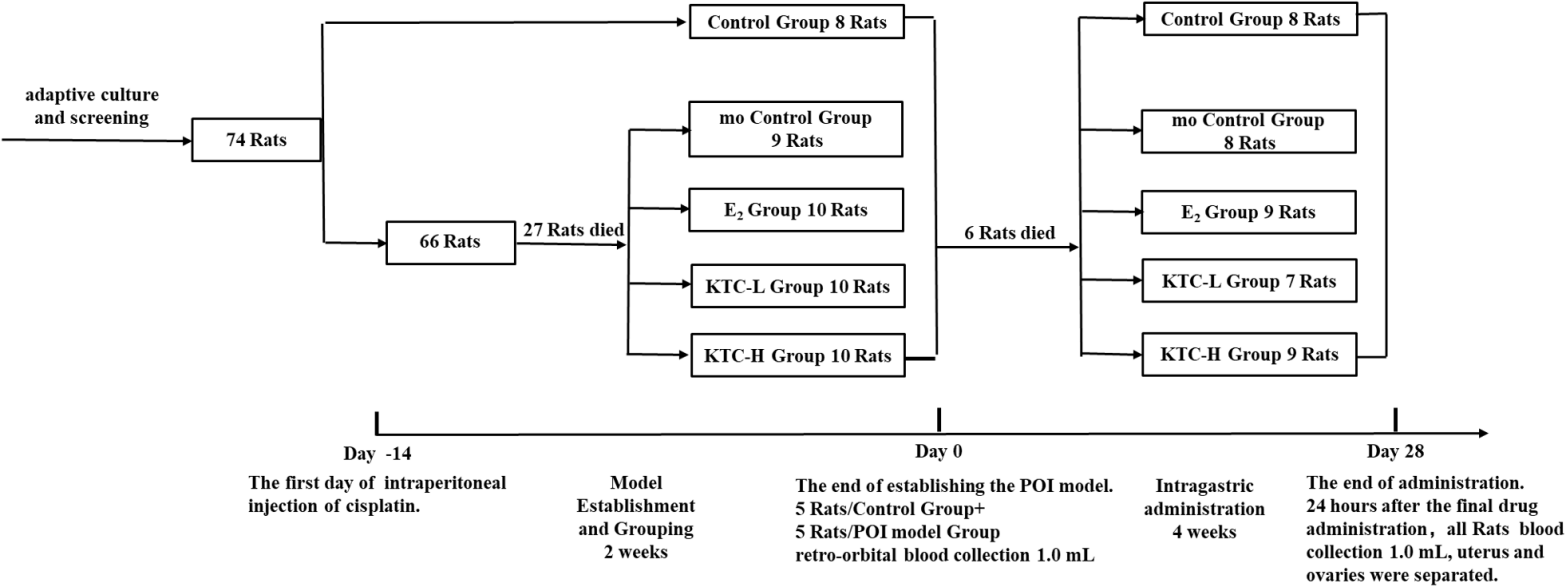
Flow chart of the experiment.

For the 28-day administration, the Control and mo Control groups were intragastrically administered with 2.0 mL of 0.9% sodium chloride daily; the E_2_ group with 2.0 mL of Estradiol suspension at a dose of 0.2mg/kg/d (Abbott Biologicals BV lot number:356933, Strength: 2mg/tablet); and the KTC-L and KTC-H groups with 2.0 mL of Kuntai capsule suspension (Guiyang Xintian Pharmaceutical Co. Ltd. lot number: 180405, Strength: 0.5g; 0.6g/kg/d, 1.8g/kg/d, respectively). Animal drug dose conversion was calculated based on the coefficient of equivalent dose transferring from rats to humans using body surface area (14) (**Figure 1** and **Table 1**).

**Table 1:** Grouping, administration, and survival of the rats.

Groups	Administration	Numbers of rats	Dose (mg/kg)	Dose (mL/kg)	Duration (weeks)	Numbers survived
Control	0.9% sodium chloride	8	–	10	4	8
mo Control	0.9% sodium chloride	9	–	10	4	8
E_2_	Estradiol suspensoid	10	0.2	10	4	9
KTC-L	Kuntai capsule suspensoid	10	600.0	10	4	7
KTC-H	Kuntai capsule suspensoid	10	1800.0	10	4	9

### 2.2 Indicators and material

#### 2.2.1 Estrous cycle

During administration, the vaginal smears of five rats in each group were evaluated between 09:00 and 10:00 using Wright’s-Giemsa stain to assess the estrous cycles.

#### 2.2.2 Blood collection and evaluation of AMH, FSH, and E_2_

After the POI model was established, five rats were randomly selected from the Control and POI model groups. Blood samples of 1.0 mL/rat were collected from the retro-orbital sinus. Twenty-four hours after the last drug administration, blood samples of 1.0 mL of all rats were collected from the abdominal aorta. Serum AMH, FSH, and E_2_ levels of the blood samples were measured by enzyme-linked immunosorbent assay (ELISA).

#### 2.2.3 Ovarian sample collection

After the final drug administration, rats were sacrificed by overdose of sodium pentobarbital (100mg/kg). Ovaries were removed immediately for further analysis, and ovarian fat pads were gently withdrawn. Bilateral ovaries were weighted with analytical balance, and ovarian coefficients were recorded.

The left ovarian tissue was fixed in 4% paraformaldehyde, dehydrated with gradient alcohol, embedded with paraffin, and sectioned for hematoxylin and eosin (HE) staining to evaluate the number of resting follicles (primordial follicles + primary follicles) and growing follicles (secondary follicles + antral follicles). The right ovarian tissue was frozen in liquid nitrogen and stored at a -80°C freezer for protein analysis of Growth differentiation factor-9 (GDF9), light chain 3 (LC3) A-II, and Beclin 1, which are relative to follicle development or autophagy.

### 2.3 Statistical analysis

The Shapiro-Wilk test was used to check if the continuous variables follow a normal distribution, and Levene’s test was used to assess the equality of variances. Variables normally distributed were shown as ‘mean ± SD’. One-way analysis of variance (ANOVA)-LSD was used for comparing group means. *P* < 0.05 was considered statistically significant.

## 3 Results

### 3.1 Estrous cycle

**Figure 2:**
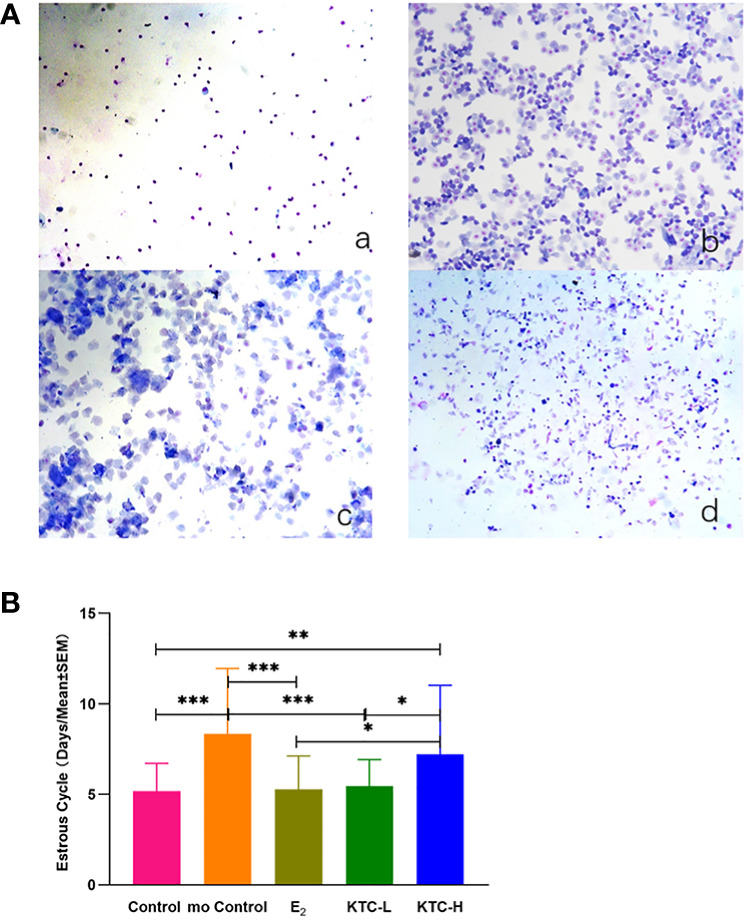
**(A)** The typical pictures of smears of vaginal cells at different stages of the estrous cycle in rats (×100). a Diestrus; b Proestrus; c Estrus; d Metestrus. **(B)** The estrous cycle in five groups. * *P*<0.05; ** *P*<0.01; *** *P*<0.001.

The typical pictures of smears of vaginal cells at different stages of the estrous cycle in rats are shown in **Figure 2A**. The estrous cycles of the Control (5.18 ± 1.54) and KTC-L groups (5.46 ± 1.48) were significantly lower than the mo Control (8.35 ± 0.60) and KTC-H groups (7.2 ± 3.83) (*P*=0.000, *P*=0.006; *P*=0.000, *P*=0.02; respectively). No statistical significance was observed when comparing the KTC-L group with the Control and E_2_ groups (*P*=0.676; 5.30 ± 1.83, *P*=0.809, respectively) (**Figure 2B**).

### 3.2 Serum E_2_, FSH, and AMH levels after establishing the POI model and final administration

**Figure 3:**
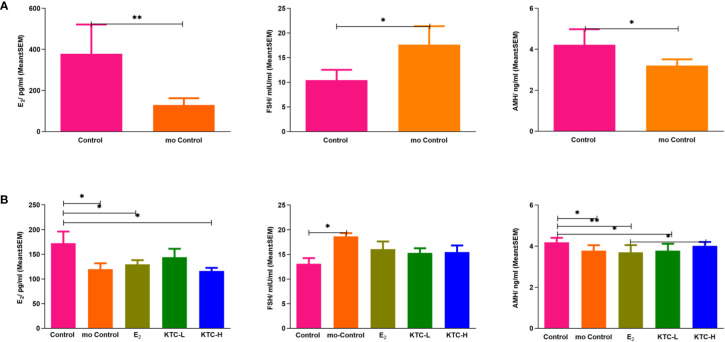
**(A)** Serum E_2_, FSH, and AMH levels after establishing the POI model. * *P*<0.05; ** *P*<0.01 **(B)** Serum E_2_, FSH, and AMH levels in the five groups after administration for 28 days. * *P*<0.05; ** *P*<0.01.

As shown in **Figure 3A**, the AMH and E_2_ levels of the POI model group were significantly lower than the Control group (4.15 ± 0.92 vs. 3.34 ± 0.42, *P*=0.044; 378.12 ± 142.77 vs. 129.19 ± 33.31, *P*=0.005), while the FSH levels of the POI model group were significantly higher (17.64 ± 3.74 vs. 10.43 ± 2.09, *P*=0.019), thus indicating the successful establishment of cisplatin-induced POI rat model.

As shown in **Figure 3B**, the E_2_ level of the Control group (172.69 ± 23.39) was significantly higher than the mo Control, E_2_, and KTC-H groups (120.12 ± 25.92, *P*=0.028; 129.76 ± 25.37, *P*=0.035; 116.36 ± 19.90, *P*=0.011, respectively). However, the E_2_ level of the KTC-L group (136.77 ± 38.78) was not significantly different from the Control group (*P*=0.082). The FSH level of the Control group (13.10 ± 2.32) was significantly lower than the mo Control group (18.65 ± 1.31, *P*=0.038) but not significantly different from the E_2_, KTC-L, and KTC-H groups (16.05 ± 4.72, *P*=0.185; 15.30 ± 2.12, *P*=0.370; 15.46 ± 3.87, *P*=0.295; respectively). The AMH level of the Control group (4.18 ± 0.22) was significantly higher than the mo Control, E_2_, and KTC-L groups (3.78 ± 0.27, 3.70 ± 0.36, 3.78 ± 0.34) (*P*=0.006, *P*=0.001, *P*=0.009), but not significantly different from the KTC-H group (4.02 ± 0.19, *P*=0.228). The AMH level of the KTC-H group was significantly higher than the E_2_ group (*P*=0.020).

### 3.3 Ovarian coefficient

**Figure 4:**
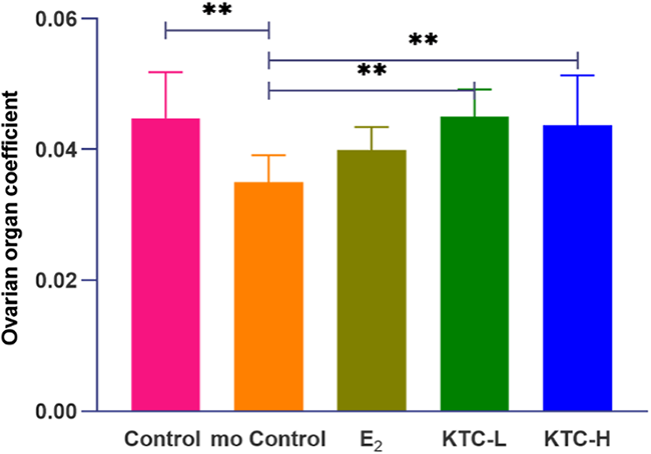
The five groups’ ovarian organ coefficient. ** *P*<0.01.

As shown in **Figure 4**, the ovarian coefficient of the mo Control group (0.035 ± 0.004) was significantly lower than the Control (0.045 ± 0.007), KTC-L (0.045 ± 0.004), and KTC-H groups (0.044 ± 0.008) (*P*=0.003, *P*=0.003, *P*=0.009, respectively), with no statistical differences observed among the latter three groups. The E_2_ group (0.040 ± 0.004) was not significantly different from the KTC-L and KTC-H groups (*P*=0.087, *P*=0.196, respectively).

### 3.4 Resting and growing follicles

**Figure 5:**
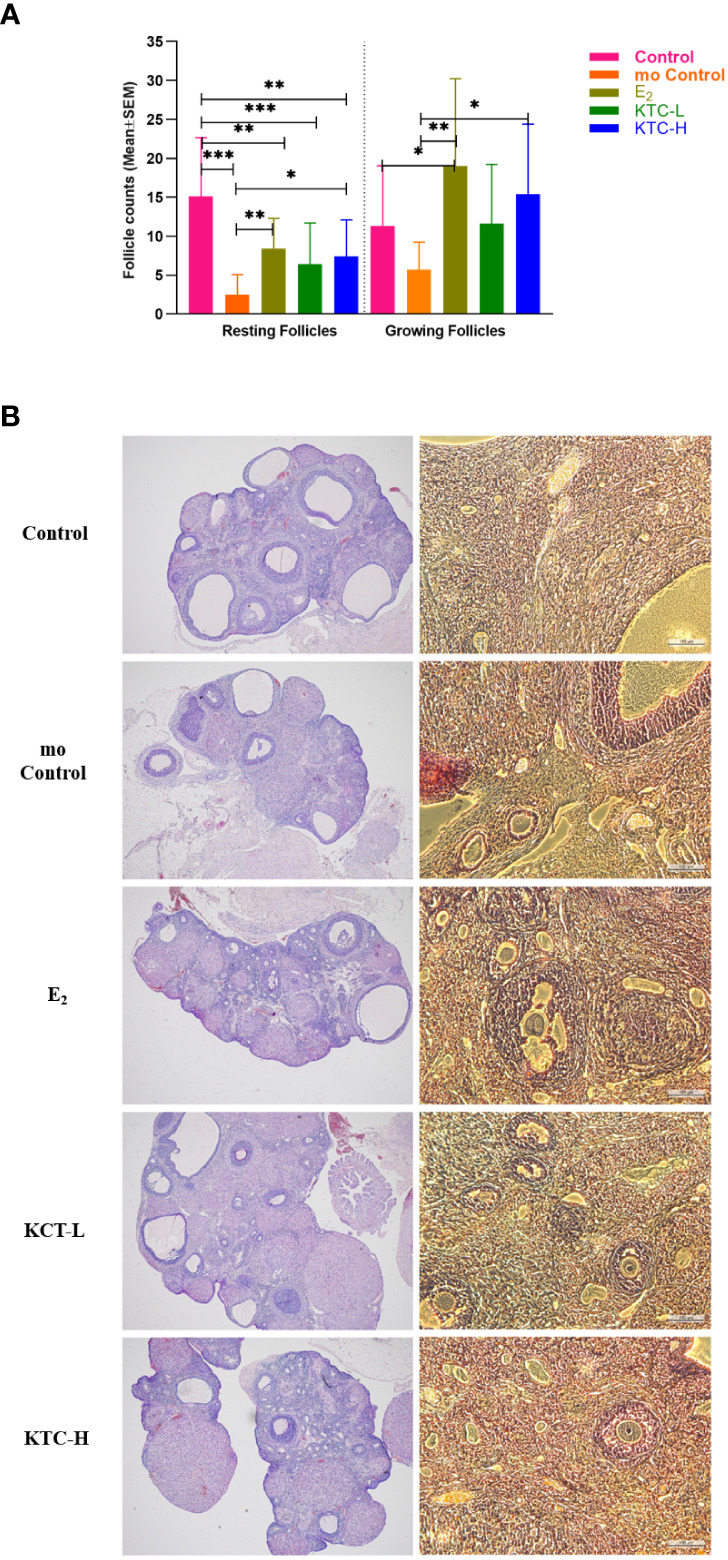
**(A)** The number of Resting and Growing follicles among the five groups is compared. **(B)** Typical HE pictures of the five groups. * *P*<0.05, ** *P*<0.01, *** *P*<0.001.

As shown in **Figure 5A**, regarding the resting follicles numbers, the Control group (15 ± 8) were significantly higher than the mo Control, E_2_, KTC-L, and KTC-H groups (3 ± 3, *P*=0.000; 8 ± 4, *P*=0.004; 6 ± 4, *P*=0.001; 7 ± 5, *P*=0.000; respectively). The E_2_, and KTC-H groups were significantly higher than the mo Control group (*P*=0.012, *P*=0.032, respectively).

Regarding the numbers of the growing follicles, the mo Control group (6 ± 4) was significantly lower than the E_2_, and KTC-H groups (19 ± 12, *P*=0.002; 15 ± 9, *P*=0.017; respectively). **Figure 5B** is the typical HE pictures of follicles of the five groups.

### 3.5 Protein expression of GDF9, LC3A-II, and Beclin 1

**Figure 6:**
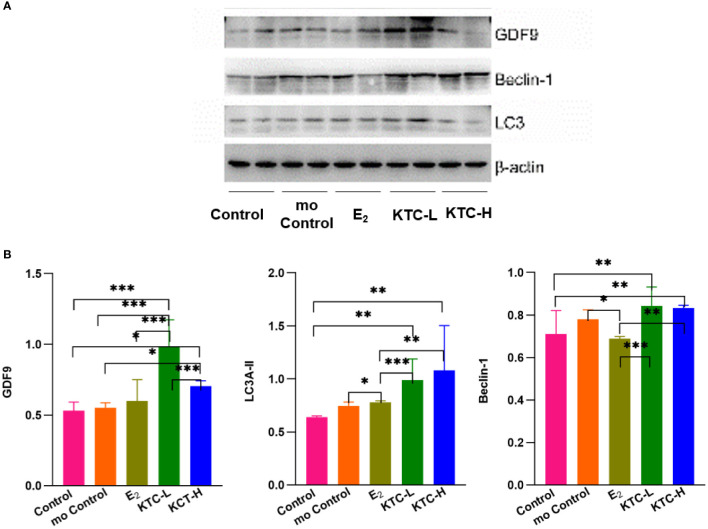
**(A)** Western blotting of GDF9, LC3A-II, Beclin-1 protein analysis. **(B)** The relative protein expression of GDF9, LC3A-II, Beclin-1 in the five groups. * *P*<0.05, ** *P*<0.01, *** *P*<0.001.

As shown in **Figure 6**, regarding the relative protein expression of GDF9, both the KTC-L (0.985 ± 0.188) and KTC-H groups (0.705 ± 0.038) were significantly higher than the Control (0.530 ± 0.061) and mo Control groups (0.551 ± 0.035) (*P*=0.000, *P*=0.000; *P*=0.013, *P*=0.027, respectively).

About the relative protein expression of LC3A-II, the KTC-H group (1.080 ± 0.424) was significantly higher than the Control, mo Control, and E_2_ groups (0.641 ± 0.010, *P*=0.001; 0.746 ± 0.035, *P*=0.011; 0.780 ± 0.013, *P*=0.021; respectively). The KTC-L group (0.988 ± 0.201) was significantly higher than the Control group (*P*=0.009).

About the relative protein expression of Beclin 1, both the KTC-H (0.832 ± 0.014) and KTC-L groups (0.843 ± 0.088) were significantly higher than the Control (0.711 ± 0.110) and E_2_ groups (0.688 ± 0.011) (*P*=0.004, *P*=0.001; *P*=0.002, *P*=0.000; respectively). The mo Control group (0.781 ± 0.044) was significantly higher than the E_2_ group (*P*=0.023).

## 4 Discussion

Cisplatin has been one of the most widely used anti-tumor drugs in clinical practice in the past 20 years. Some scholars have confirmed that Cisplatin can induce cell apoptosis in the ovary and tissue necrosis, leading to POI (15). This study used Cisplatin to establish a reliable model of POI rats. The model was then administered with different doses of Kuntai capsule to observe its effect on rats’ ovarian function and explore ways of preserving ovarian function in young female patients receiving chemotherapy.

### 4.1 Follicle development

GDF9 is a member of the Transforming Growth Factor β (TGFβ) family. In addition to promoting pre-antral follicles growth, antral follicles growth, and granulosa cells proliferation, GDF9 also stimulates the granulosa cells’ basal secretion of progesterone and Estradiol of small antral follicles of female rats (16–19). In this study, GDF9 expression in both Kuntai groups was significantly higher than in the Control and mo Control groups. This indicates that Kuntai capsule has a positive effect on the development of granulosa cells in small follicles, which is consistent with the results of follicle counts.

### 4.2 Autophagy

Microtubule-associated protein 1A/1B-LC3 is a soluble protein widely distributed in mammalian tissues and cultured cells. The expression of LC3A-II in the mammalian ovaries is relatively higher than other isoforms, thus making it a suitable marker for monitoring autophagy (20–24).

The results of this study revealed the expression of LC3A-II protein in the following descending order: KTC-H > KTC-L > E_2_ > mo Control > Control. In other words, all four POI model groups had a higher LC3A-II expression than the Control group, with the KTC-H and KTC-L groups showing the most apparent autophagic responses.

The Beclin 1 gene is the mammalian ortholog of yeast autophagy-related gene-6 (ATG6) and the most widely studied autophagy-related gene. Beclin 1 acts as a platform for binding with proteins like Phosphoinositide 3-kinase (PI3KC3), ultraviolet radiation resistance-associated gene (UVRAG), activating molecule in Beclin 1-regulated autophagy (Ambra1), and Bax interacting factor-1 (Bif-1) to promote autophagy (25–29).

In this study, the expression of Beclin-1 is in the following descending order: KTC-L > KTC-H > mo Control > Control > E_2_. The results showed that although autophagy is common among the five groups, Beclin-1 expression in both the KTC-L and KTC-H groups was significantly higher, indicating that Kuntai Capsule induces stronger autophagy than the other administration.

Autophagy plays dual roles in cell survival and death by inhibiting or partnering with apoptosis. Autophagy helps the body eliminate cancerous cells, and damaged organelles facilitate cellular adaptation to the changing internal and external environment and promote cell survival by delaying aging and cell death (30, 31).

### 4.3 Traditional Chinese medicine network pharmacology and Kuntai capsule

Unlike modern medicine, which has a single active compound, Traditional Chinese Medicine integrates many plants into formulae of compatible ingredients. Each ingredient contains various compounds, with complex interactions among these compounds forming a network. This also gave rise to the development of the discipline ‘Traditional Chinese Medicine Network Pharmacology’ (32–35), which updates the research paradigm from the current ‘one target, one drug’ mode to a new ‘network target, multi-components mode’ (36).

Research on the network pharmacology of Kuntai Capsule has been carried out recently. Kuntai Capsule consists of 6 herb components: Rehmannia glutinosa, Coptis, Paeonia, Scutellaria, Equus asinus, and Poria cocos. Li DX (37) utilized the Traditional Chinese Medicine Systems Pharmacology Database and Analysis Platform (TCMSP) to find out 21 active compounds corresponding to the six herb components, which are mainly flavonoids and alkaloids, including verbascoside, 5-hydroxymethylfurfural, baicalein, wogonin, skullcapflavone II, berberine, columbamine, magnoflorine, jatrorrhizine, and palmatine, etc. Wang LJ and Chen YM (38, 39) did further research and acquired the corresponding 186 gene targets in the cells of the human body of these 21 compounds *via* TCM-mesh (http://mesh.tcm.microbioinformatics.org) and COSMIC (http://cancer.sanger.ac.uk/cosmic). These gene targets are highly enriched in the central nervous system, breast, endometrium, and ovary. This result indicates that the Kuntai capsule can treat related diseases caused by ovarian insufficiency. Besides, Kyoto Encyclopedia of Genes and Genomes (KEGG) results found that these targets were involved in the PI3K-Akt, mTOR, and insulin signaling pathways, which participate in follicular development in the follicle recruitment stage (38–40). Signaling pathways may become one of the directions in further research.

### 4.4 Mortality rate in establishing the POI rat model

Several studies used different doses of cisplatin to establish the POI rat model, the median lethal dose (LD_50_) of cisplatin in rats ranging from 6 to 20 mg/kg (41–43). A dose less than 6 mg/kg can reduce death, but the POI model would not be successful enough to carry out further experiments; doses higher than 6 mg/kg would ensure a higher success of modeling, but it would have adverse effects such as decreased appetite, weight loss, and myelosuppression, and especially a higher risk of death (9). In a study (10) comparing lower doses of cisplatin (1.5 mg/kg, 2.0 mg/kg, or 2.5 mg/kg once a day for five consecutive days) with higher dose of cisplatin (4 mg/kg on Day 1 and 6 mg/kg on Day 8), it was concluded that the lower dose regimes were not effective to establish the POI model by examining the body mass, follicle count, and serum AMH.Therefore, in our pre-experiment study, we did not adopt the lower-dose cisplatin method to establish the POI model and followed the higher-dose modeling method (9).

Our mortality rate, at 41%, was about in the range of other studies as far as mortality rates have been reported (41–43). Contrary to those studies that do not mention their mortality rate in the modeling process, we intended to list out the number of rats that died in the modeling process to act as a reference for researchers planning to use cisplatin-induced POI rat models and a reminder that other means need to be explored to reduce mortality rate while ensuring the modeling effectiveness, such as caudal injection of cisplatin or subcutaneous injection of saline to reduce dehydration. Our intention was to stress the need and the possibilities to reduce the mortality due to the toxic cisplatin.

## 5 Limitation

There are still limitations to this study. The indicators in our research are not comprehensive enough. In further research, we will add TUNEL staining to measure apoptosis, Western blot analysis of SQSTM1/P62 to ensure the role of Kuntai Capsule-induced autophagy, perform GDF9, LC3A-II, and Beclin1 immunohistochemistry and immunofluorescence stainings, and culture primary ovarian granulosa cells *in vitro* to further ensure the role of Kuntai Capsule in E_2_ secretion and activation of autophagy.

## 6 Conclusion

Traditional Chinese medicine Kuntai capsule can improve POI rats’ estrous cycle and ovarian coefficient, increase the numbers of resting and growing follicles, and up-regulate the protein expression of GDF9, LC3A-II, and Beclin 1 of the ovary. Further research needs to be done to have an in-depth understanding of the mechanisms involved.

## Data availability statement

The data presented in the study are deposited in the Figshare repository, accession number: https://doi.org/10.6084/m9.figshare.21896502.

## Ethics statement

The animal study was reviewed and approved by The Ethics Committee of Capital Medical University (ethics code: AEEI-2018-163).

## Author contributions

All authors qualify for authorship by contributing substantially to this article. SL: Performed the experiment, wrote the original draft, and revised the manuscript. XR: Project leader, project supervisor, and revised the first draft. AM: Experimental supervision, interpretation of results, and article revision. All authors contributed to the article and approved the submitted version.
